# Impact of Viral Lysis on the Composition of Bacterial Communities and Dissolved Organic Matter in Deep-Sea Sediments

**DOI:** 10.3390/v12090922

**Published:** 2020-08-22

**Authors:** Mara E. Heinrichs, Dennis A. Tebbe, Bernd Wemheuer, Jutta Niggemann, Bert Engelen

**Affiliations:** 1 Institute for Chemistry and Biology of the Marine Environment, Carl von Ossietzky University of Oldenburg, 26129 Oldenburg, Germany; mara.elena.heinrichs@uni-oldenburg.de (M.E.H.); dennis.alexander.tebbe@uni-oldenburg.de (D.A.T.); jutta.niggemann@uni-oldenburg.de (J.N.); 2 Genomic and Applied Microbiology and Göttingen Genomics Laboratory, Institute of Microbiology and Genetics, University of Göttingen, 37073 Göttingen, Germany; bernd.wemheuer@biologie.uni-goettingen.de

**Keywords:** virus-induction, mitomycin C, killing the winner, Bering Sea, prophages

## Abstract

Viral lysis is a main mortality factor for bacteria in deep-sea sediments, leading to changing microbial community structures and the release of cellular components to the environment. Nature and fate of these compounds and the role of viruses for microbial diversity is largely unknown. We investigated the effect of viruses on the composition of bacterial communities and the pool of dissolved organic matter (DOM) by setting up virus-induction experiments using mitomycin C with sediments from the seafloor of the Bering Sea. At the sediment surface, no substantial prophage induction was detected, while incubations from 20 cm below seafloor showed a doubling of the virus-to-cell ratio. Ultra-high resolution mass spectrometry revealed an imprint of cell lysis on the molecular composition of DOM, showing an increase of molecular formulas typical for common biomolecules. More than 50% of these compounds were removed or transformed during incubation. The remaining material potentially contributed to the pool of refractory DOM. Next generation sequencing of the bacterial communities from the induction experiment showed a stable composition over time. In contrast, in the non-treated controls the abundance of dominant taxa (e.g., Gammaproteobacteria) increased at the expense of less abundant phyla. Thus, we conclude that viral lysis was an important driver in sustaining bacterial diversity, consistent with the “killing the winner” model.

## 1. Introduction

Deep-sea sediments roughly harbor a third of the global prokaryotic biomass and represent one of the largest organic carbon reservoirs on Earth [1,2]. Prokaryotes thriving in this environment play a dominant role in benthic carbon turnover and the regeneration of nutrients [3]. Another important component for carbon cycling in deep-sea sediments are benthic viruses as they significantly influence bacterial communities and associated biogeochemical cycles [4,5]. Virus-mediated cell lysis is considered to be the main cause of prokaryotic mortality, resulting in the reduction of up to 80% of total heterotrophic microbial production on a global scale [4,6].

The majority of infections by viruses infecting bacteria (bacteriophages) are either lytic or lysogenic. A lytic infection ultimately leads to cell lysis, which is accompanied by the release of newly produced virus particles and cell debris into the environment [7]. In contrast to lysing their host immediately, temperate viruses can integrate into the host’s genome, where they replicate as prophages during cell division. Prophages were experimentally and bioinformatically identified in 40–70% of all available prokaryotic genomes [8,9]. They remain dormant within the host until the lytic cycle is induced. The induction can happen spontaneously [10] or by physical stress and chemical inducing agents [11]. The virus-mediated generation of dissolved organic matter (vDOM) provides substrates for indigenous prokaryotes [12] and represents a shortcut in the marine foodweb, the so-called “viral shunt” [7]. It is estimated that viral lysis accounts for the annual release of up to 0.63 Gt of organic carbon in marine sediments [4]. So far, it is not known how these lysis products and the subsequent microbial transformations impact the composition of the pool of DOM in deep-sea sediments.

In general, DOM is a highly complex mixture of thousands of different molecules [13]. With respect to reactivity, DOM can roughly be classified in two main fractions [14]: biologically available molecules (“labile”) as resource for heterotrophic bacteria, ultimately fueling the marine food web [15], and refractory compounds, resisting microbial utilization [16]. In the world’s oceans, organic matter produced in the photic zone is intensively processed on its way down to the deep sea and becomes increasingly resistant to microbial degradation with depth [17]. Consequently, prokaryotes at the seafloor have to cope with organic molecules that are rather difficult to degrade [18]. Thus, the virus-mediated release of highly bioavailable cell material potentially plays a crucial role in sustaining heterotrophic life in deep-sea surface sediments [4]. However, not all lysis products are labile, some can also be less biodegradable or difficult to break down [12]. The remnants of cytoplasmic and structural cell components [19] for example might accumulate and contribute to the persistent pool of refractory DOM [14].

The available substrate spectrum can further be modified by viruses, as they shape bacterial community structures, e.g., by the selective removal of individual groups [20]. Fast-growing and more abundant prokaryotes generally experience a stronger predation pressure due to higher production and encounter-rates of viruses. The density-dependent relationship between viruses and their hosts can lead to selective killing of numerically dominant, highly competitive taxa. This virus-mediated community shift is known as the “killing the winner” model [21]. The mechanism is considered a key process in maintaining prokaryotic evenness and richness [22]. However, viruses were reported to have variable effects on the phylogenetic composition of bacteria [23,24]. It is currently not known how viruses regulate bacterial communities in deep-sea sediments.

In order to understand the role of deep-sea viruses in shaping the structure and diversity of bacterial communities as well as composition and reactivity of the DOM pool, we performed a prophage induction experiment [25], which offers the opportunity to study the nature and fate of vDOM and the associated bacterial community shift. Sediments were retrieved from the highly productive Bering Sea. The sampling site is characterized by an estimated primary production of >170 g C m^−2^ y^−1^ [26] and an efficient biological carbon pump [27], leading to relatively high organic carbon burial and microbial mediated remineralization rates [28]. Since virus-mediated bacterial mortality was found to be enhanced in more eutrophic systems [29], we expected a major impact of viruses on this deep-sea ecosystem. In our experiment, sediment slurries were treated with the antibiotic mitomycin C to stimulate prophage induction. Prokaryotic and viral abundances as well as concentrations of labile organic compounds (amino acids and carbohydrates) were monitored over the course of 55 days. We used Fourier transform ion cyclotron resonance mass spectrometry (FT-ICR-MS) to characterize the molecular composition of vDOM in great detail [30]. Changes of bacterial community structures in response to the viral activity were identified by Illumina sequencing of 16S rRNA genes and transcripts. We hypothesized that (i) the induction of prophages and subsequent cell lysis leads to a transient increase of labile organic compounds within the DOM pool, ultimately leaving behind more refractory components, and that (ii) selected bacterial community members are more susceptible to viral lysis, while others take advantage of the reduced substrate competition and the viral shunt, e.g., by increasing in abundances, leading to an overall shift of bacterial groups. As an outcome of this experiment, we concluded that viruses had a clear impact on indigenous bacterial populations and the DOM pool, depending on the sediment depth.

## 2. Materials and Methods

### 2.1. Origin of Sediments

Our sampling site exhibited a water depth of 3300 m and was located near the highly productive continental shelf break of the Bering Sea (178° 55,755′ W, 58° 54,471′ N). Sediments were retrieved during RV Sonne expedition SO248 in May 2016 by a multicorer (Octopus, Kiel, Germany). The time for core recovery was about three hours, limiting the deployment of several multicorers due to ship time restrictions. Subsamples were taken from two different depths, i.e., the sediment surface (0–2 cm below seafloor (cmbsf)) and 20–22 cmbsf. For each depth, two times 400 cm^3^ of sediment was pooled from seven cores. The sediment was kept in sterilized 1-L glass bottles at 4 °C in the dark until the start of the experiment in the home laboratory. Biogeochemical background data of the sediments were analyzed as described in Pohlner et al. [31] (Supplementary Table S1).

### 2.2. Experimental Setup and Subsampling Procedure

The pooled sediment was mixed with the same amount of sterile artificial seawater [32] two weeks after sampling to create slurries. The limited amount of sediment did not allow setups in replicates. To induce the prophages, one slurry of each depth was supplemented with mitomycin C (“treatment”) (1 µg mL^−1^ final concentration, Roth, Karlsruhe, Germany). The other slurry served as control. The bottles were incubated at an in situ temperature of 4 °C in the dark. As both investigated sediment layers were oxic, the slurries were aerated and stirred on each sampling day.

Samples for total cell counts (TCCs) (0.5 mL of slurry) and virus-like particle (VLP) counts (1 mL of slurry) were taken daily in the first 14 days to follow a potential induction event and after 55 days of incubation. Main sampling time points were defined according to the development of TCCs and VLPs: day 0 = starting point to record the initial conditions, day 6 = potential prophage induction, day 14 = potential bacterial response in growth, day 55 = end point to record system stabilization. On every main time point, 165 mL of slurry were taken from each bottle.

Samples for cell counting were fixed with 3% glutaraldehyde for 2 h at room temperature [33]. Afterwards, the samples were spinned down, washed twice with 1 mL TAE-buffer (pH 7.4) and stored in TAE:EtOH (1:1) at −20 °C until analysis. Samples for virus counts were immediately stored at −80 °C. Pore water was taken on the main sampling days by using rhizons (pore size 0.12–0.18 µm, Rhizosphere, Wageningen, The Netherlands). For the analysis of amino acid and sugar concentrations pore water was stored at −20 °C. For the quantification of dissolved organic carbon (DOC) and characterization of the DOM molecular composition, pore water was stored in pre-combusted (400 °C, 4 h) glass vials, acidified to pH 2 with 25% HCl (p.a.) and stored at 4 °C in the dark. Furthermore, aliquots of the slurries were frozen at −80 °C for DNA and RNA extraction to investigate the bacterial community compositions.

### 2.3. Enumeration of Prokaryotic Cells and Virus-Like Particles

TCCs were performed using epifluorescence microscopy, following a modified protocol by Lunau et al. [33] as described previously [31]. Briefly, sub-samples of the slurries were sonicated three times for 1 min, interrupted by a cooling step for 1 min. The supernatant was transferred, diluted with TAE: EtOH (1:1) and spread on a microscope slide to dry. After staining with a Sybr Green I solution, 10 to 20 microscopic fields were randomly selected and at least 300 cells were counted at a magnification of 400× using an epifluorescence microscope (Zeiss, Oberkochen, Germany).

Prior to counting, VLPs were extracted from slurries according to Danovaro et al. [34], with slight modifications as described in the following. Samples for quantification were fixed with 0.5% glutaraldehyde (EM-grade) after thawing and immediately processed to avoid loss of VLPs. Three cycles of extraction (1 min sonication, followed by 30 s cooling on ice) were performed. The resulting extracts were pooled and pre-filtered through a filter with 0.45 µm pore size. Immediately after extraction, VLPs were quantified via epifluorescence microscopy [35]. Therefore, aliquots of the extract were filtered through 0.02 µm Anodisc 25 membrane filters (Whatman, Maidstone, UK) while applying a negative pressure of -50 mbar. After staining with Sybr Green I for 15 min in the dark, the rinsed filter was placed on an object slide and embedded in freshly prepared mounting buffer [35]. At least 300 VLPs were counted in randomly selected fields using a BX51 microscope (Olympus, Tokyo, Japan) with a 100× objective (UPLan FL N, Olympus) and an EGFP HC filter set (Semrock, New York, NY, USA).

### 2.4. Nucleic Acid Extraction and Sequencing

Changes in the total and potentially active bacterial communities during the experiment were assessed by Illumina sequencing of 16S rRNA amplicons, generated from 16S rRNA genes and transcripts as described previously [31]. Briefly, DNA was extracted from 0.5 mL slurry using the DNeasy PowerSoil kit (Qiagen, Hilden, Germany) according to the manufacturer’s instructions. RNA extractions of 0.5 mL slurry were performed by the Allprep DNA/RNA Mini Kit (Qiagen) following the provided instructions with modifications as described by Pohlner et al. [31]. The concentration and purity of the nucleic acid extracts were evaluated spectrophotometrically (NanoDrop 2000c, Thermo Fisher Scientific, Waltham, MA, USA) and stored at −20 °C until further analysis.

For sequencing, resulting DNA extracts were treated with RNase A and the GeneRead Size Selection kit and RNA extracts with Turbo DNase, before transcription into cDNA by SuperScript III reverse transcriptase (Thermo Fisher Scientific) using the reverse primer S-D-Bact-0785-a-A-21 without MiSeq adapter (5′-GAC TAC HVG GGT ATC TAA TCC-3′) [36]. Three independent rounds of amplification of DNA and cDNA via PCR were performed using Phusion polymerase and the primer pair S-D-Bact-0341-b-S-17 (5′-CCT ACG GGN GGC WGC AG-3′) and S-D-Bact-0785-a-A-21 (5′-GAC TAC HVG GGT ATC TAA TCC-3′) [36] with Illumina NexTera adapters. The independently generated 16S rRNA amplicon libraries were pooled, barcoded with the NexTera XT-Index Kit (Illumina, San Diego, CA, USA) and Kapa HIFI Hot Start polymerase (Kapa Biosystems, Wilmington, MA, USA) and sequenced at the Goettingen Genomics Laboratory on an Illumina MiSeq System using the MiSeq Reagent kit v3 (paired end 2 × 300 bp; Illumina).

### 2.5. Analysis of the Illumina Data Sets

The resulting data sets of 16S rRNA genes and transcripts were processed according to Granzow et al. [37] as described previously [31]. After quality filtering and merging paired-end reads using Trimmomatic version 0.32 [38] and USEARCH version 8.0.1623 [39], respectively, the remaining sequences were combined and clustered in operational taxonomic units (OTUs) at 3% genetic divergence using the UPARSE algorithm. After removal of singletons, remaining chimeric sequences were removed using the Uchime algorithm in reference mode [40]. OTU sequences were then taxonomically classified using QIIME [41] by BLAST alignment against the SILVA database (SILVA SSURef 128 NR) and the QIIME release of the UNITE database (version 7.1), respectively. All non-bacterial OTUs were removed. Finally, processed sequences were mapped on OTU sequences to calculate the distribution and abundance of each OTU in every sample. Barplots of the relative abundances of bacterial taxa were generated using R (version 3.6.2) [42] and the package “phyloseq” [43]. Phyla that exhibited relative abundances below 1% for the whole experimental period were summarized to the group “Others”. Species richness, Shannon diversity and Pielou’s evenness were calculated with the vegan package [44] in R, based on rarefied data of the OTU table (11,000 reads per sample, 30 bootstraps). Sequence data were deposited in the sequence read archive of the National Center for Biotechnology Institute under the accession numbers SRR11696610-SRR11696645 (BioProject PRJNA630567).

### 2.6. Quantification of Amino Acids, Carbohydrates and Dissolved Organic Carbon

Changes in the concentrations of the ten most polar dissolved free amino acids (DFAA) and total dissolved hydrolyzed amino acids (THDAA) were determined via reversed-phase high-performance liquid chromatography after ortho-phthaldialdehyde precolumn derivatization [45] using an external standard [46]. Samples were run on an Agilent Technologies 1200 system using Zorbax Eclipse columns (guard column: XDB-C18, 4.6 × 12.5 mm, analytical column: XDB-C18, 4.6 × 150 mm; Agilent Technologies, Santa Clara, CA, USA). Dissolved combined amino acids (DCAA) were calculated by subtracting DFAA from THDAA concentrations. Detection limit of the method was 2 nM and 5 nM for DFAA and THDAA, respectively.

Concentrations of dissolved free neutral monosaccharides (DFCHO) and dissolved combined monosaccharides (DCCHO) were determined via high-performance anion exchange chromatography with pulsed amperometric detection [47]. Samples were analyzed after desalting on a Dionex ICS-5000+ instrument using a CarboPac PA10 column (Thermo Fisher Scientific) and 18 mM NaOH as eluent. Detection limit of this method was 10 nM.

DOC concentrations were quantified by high temperature catalytic oxidation using a Shimadzu TOC-VCPH total organic carbon analyzer (Kyoto, Japan) equipped with an ASI-V autosampler and a TNM-1 module. Analytical precision and trueness was checked by analyzing deep-sea reference material (DA Hansell, University of Miami, USA) and was <5%.

### 2.7. Molecular Characterization of Dissolved Organic Matter

Prior to non-targeted FT-ICR-MS analysis, DOM was concentrated from 30 mL of filtered and acidified pore water [48] using solid-phase extraction on 100 mg Bond Elut PPL cartridges with a styrene divinyl benzene polymer (Agilent Technologies). Solid-phase extractable DOM was eluted with 1 mL of methanol (HPLC-grade) and stored at –20 °C until mass spectrometric analysis. Extraction efficiencies could not be determined due to the small extract volume and low DOC concentrations.

The DOM extracts were mixed with ultrapure water and methanol (ULC/MS grade, Biosolve, Valkenswaard, The Netherlands), aiming for a similar total signal intensity for the analysis on a 15 Tesla solariX FT-ICR-MS (Bruker Daltonik, Bremen, Germany). Instrument settings and the calibration procedure are described in Seidel et al. [49] with slight modifications as depicted below. The samples were ionized by electrospray ionization in negative mode with a capillary voltage set at 4.5 kV, ions were accumulated in a hexapole for 0.1 s, and 200 scans were recorded for each mass spectrum in broadband mode using 8 megaword data sets in a mass window of 92.1 to 2000 Da. All samples were analyzed in triplicates and random order. To test instrument’s stability and reproducibility, reference DOM material of North Equatorial Pacific Intermediate Water (NEqPIW) was run at the beginning, in the middle and at the end of each measurement day. The mass error of the calibration was <0.07 ppm for all samples. Molecular formulas were assigned to detected masses with a minimum signal-to-noise ratio of 4 by using in-house MatLab routines, following the criteria published in Seidel et al. [49] and Rossel et al. [50]. The identified molecular formulas were categorized in compound groups, based on their elemental composition and elemental ratios of H/C and O/C. Therefore, the modified aromaticity index (AI_mod_) and double bond equivalents (DBE) were calculated [51]. Aromatics were indicated by an AI_mod_ > 0.5, highly unsaturated compounds by an AI_mod_ ≤ 0.5 and H/C < 1.5, unsaturated compounds by H/C ≥ 1.5 and DBE ≠ 0, saturated compounds by DBE = 0 and potential proteins by H/C ≥ 1.5, DBE ≠ 0 and N > 0. Several different isomers may exist for each molecular formula [52]; therefore, group assignments are not unambiguous.

To remove analytical noise, potential contaminations and rare, not reproducible peaks from the data set, only mass peaks that were detected at least 3 times were included in further data analysis [53]. In addition, the minimum detection limit was increased by 5% to account for slight differences in detection limit of individual spectra. This procedure may exclude compounds with low signal intensities that fall below the threshold, but most importantly excludes potential outliers. Additionally, peaks were removed, when signal-to-noise ratios of peaks detected in blanks exceeded respective signal-to-noise ratios in samples more than 20 times, or were present in at least 7 of the 10 analyzed blank samples. Double assigned molecular formulas were removed by deleting formulas with following combinations of heteroatoms in the indicated order: NSP, N_2_S, N_3_S, N_4_S, N_2_P, N_3_P, N_4_P, NS_2_, and S_2_P. Remaining double assignments were deleted from the data set. Six samples of the analytical replicates were considered as obvious outliers and manually removed from the data set. Finally, the signal intensity of each mass peak with assigned molecular formula was normalized with the sum intensity of all peaks remaining in the sample after quality control.

### 2.8. Statistical Analyses of DOM Data Set

All statistical analyses were performed on normalized data in R (version 3.6.2). The package ggplot2 (version 3.2.1) [54] was used for graphics. Normalized DOM data were Hellinger-transformed for subsequent principal coordinate analysis (PCoA) [55]. PCoA was performed on Bray–Curtis dissimilarity matrices [56], calculated using the package stats (version 3.6.2). Selected environmental vectors were fitted to the PCoA scores using the “envfit” function of the vegan package (version 2.5-6) [44] to assess the contribution of the individual parameters to the variability among the DOM samples. The environmental data included all parameters analyzed during the experiment as well as DOM compound groups described above. Spearman rank correlations were calculated between single molecular formula relative intensities and environmental parameters (9999 permutations). Only selected correlations with *p* < 0.001 were included in the analysis. Furthermore, van Krevelen diagrams were used to visually characterize the nature and on-going transformations of vDOM during the incubation period [57]. Therefore, only peaks that were present in all replicates were considered and means of relative intensities of the replicates were calculated.

## 3. Results

### 3.1. The Virus-to-Cell Ratio Indicates Prophage Induction at 20 cmbsf

**Figure 1:**
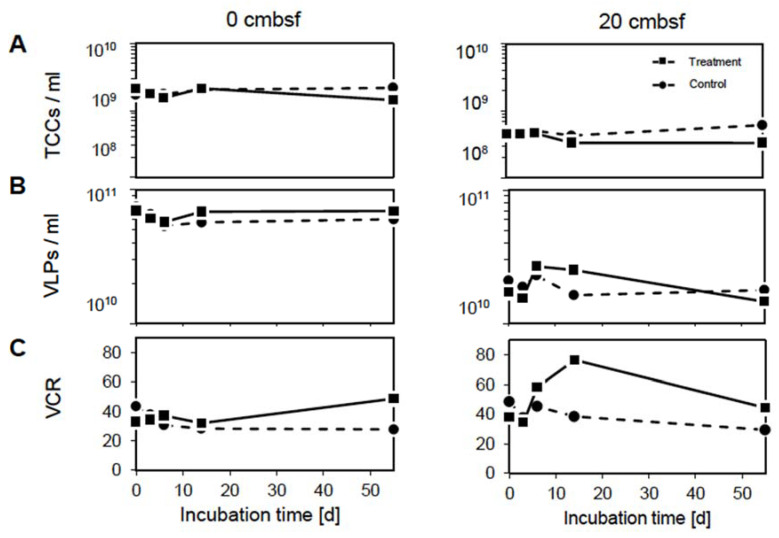
Changes in (**A**) prokaryotic and (**B**) virus abundances as well as (**C**) virus-to-cell ratio dynamics over the course of the experiment in the 0 cmbsf (left column) and 20 cmbsf incubations (right column). The mitomycin C treated incubations are displayed by the solid line and squares, the non-treated controls by the dashed line and circles.

Prophage-induction experiments with mitomycin C were performed on deep-sea sediment slurries from 0 and 20 cmbsf to assess the impact of viruses on the composition of benthic prokaryotic communities and the dissolved organic matter pool. For all quantifications of prokaryotic and virus abundances, more pronounced differences between the mitomycin C treated slurry and the non-treated control were observed in the incubations from 20 cmbsf. In general, total cell counts (TCCs) of the surface incubations were higher than those of the 20 cmbsf slurries, exhibiting 1–3 × 10^9^ and 3–6 × 10^8^ cells mL^−1^, respectively (Figure 1A). At both depths, cell numbers in the treatments were slightly lower than in the corresponding controls, most visibly at the end of the experiment. Following the TCCs, virus-like particles (VLPs) were also higher in the surface slurries compared to the 20 cmbsf incubations (Figure 1B), exceeding TCCs by one and two orders of magnitude in the surface and 20 cmbsf samples, respectively. A slight increase in VLPs was observed for the treatment compared to the controls between day 6 and day 14. Overall, the number of VLPs remained on a similar level for all incubations during the course of the experiment (5–8 × 10^10^ and 2–3 × 10^10^ VLPs mL^−1^). The increase of VLPs was again more pronounced in the 20 cmbsf treatment compared to the control. The virus-to-microbial-cell-ratio (VCR) was generally lower in the surface slurries than in the 20 cmbsf incubations (Figure 1C). A strong increase of the VCR in the treatment from 20 cmbsf was observed between day 3 and day 14. Here, the VCR more than doubled from 34 to 77, which indicates a successful prophage induction in the 20 cmbsf treatment.

### 3.2. Prophage Induction Affects the DOM Pool

**Figure 2:**
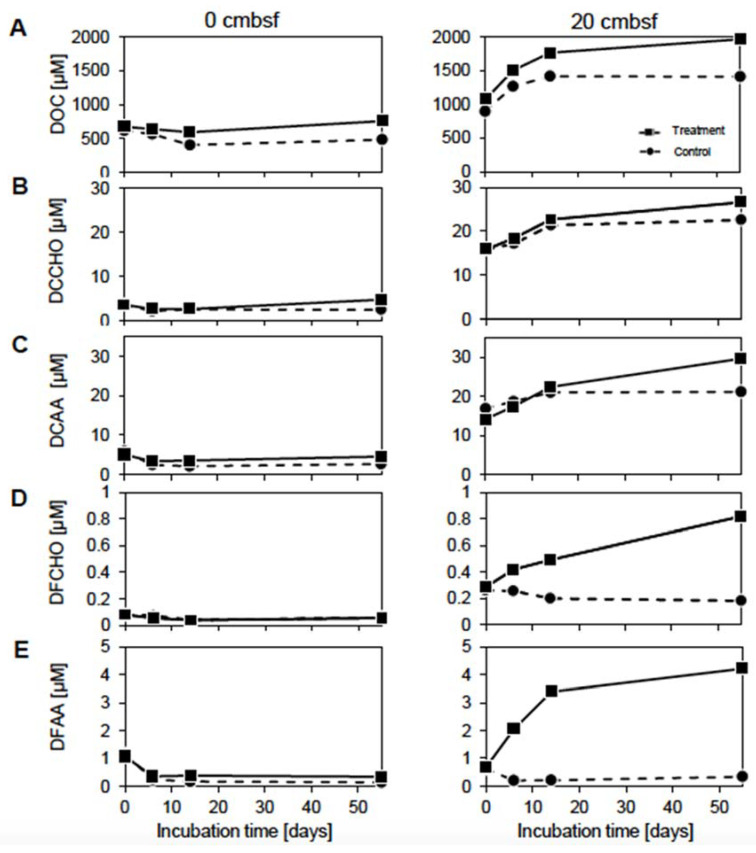
Dynamics of selected organic carbon compounds: (**A**) DOC, (**B**) dissolved combined carbohydrate, (**C**) dissolved combined amino acid, (**D**) dissolved free carbohydrate and (**E**) dissolved free amino acid concentrations. Lleft column 0 cmbsf and right column 20 cmbsf slurries. The mitomycin C treated incubations are displayed by the solid line and squares, the non-treated controls by the dashed line and circles.

To obtain a first estimate how the organic carbon pool was affected by the mitomycin C treatment, dissolved organic carbon (DOC), amino acid and carbohydrate concentrations were measured over the course of the experiment. The initial concentrations of these compounds in the 20 cmbsf incubations exceeded those of the surface, only initial concentrations of dissolved free amino acids (DFAA) were slightly higher in the surface slurries (Figure 2). In general, concentrations in the treatments were higher than in the corresponding controls. In the surface slurries, concentrations remained constantly low throughout the experiment for both, treatment and control. Contrarily, concentrations of DOC and the more complex compounds dissolved combined monosaccharides (DCCHO) and dissolved combined amino acids (DCAA) (Figure 2A–C) increased by 20–56% in the 20 cmbsf slurries, which was more pronounced in the treatments. Interestingly, dissolved free monosaccharides (DFCHO) and DFAA (Figure 2D,E) showed increasing concentrations in the treatment, only.

**Figure 3:**
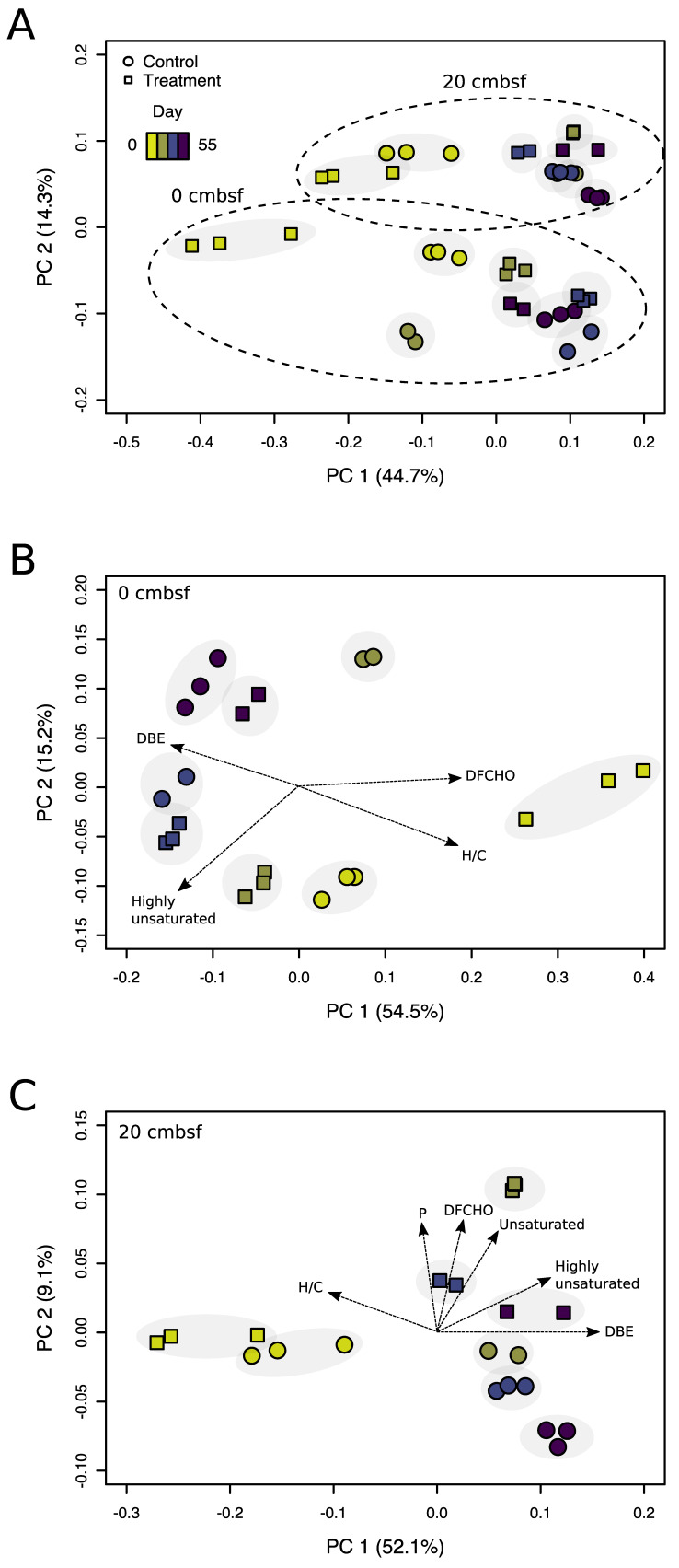
Principal coordinate analysis based on Bray-Curtis dissimilarities of relative peak intensities. Calculated for (**A**) the samples of both depths, (**B**) samples from the 0 cmbsf and (**C**) 20 cmbsf incubations, color-coded by the main sampling time points (day 0, 6, 14, and 55). The explained variance for the data set is given for the first two major axes of variation. Associated analytical replicates are marked by the gray background. Mitomycin C treated samples are displayed by squares and non-treated controls by circles. Molecular DOM parameters (black arrows) are fitted to the ordination.

Overall, the processing of the DOM data set resulted in a reduction from 837,316 detected peaks with 97,627 assigned molecular formulas to 33,373 individual mass peaks, among them 20,112 peaks with identified molecular formulas. In order to assess changes in the molecular composition of DOM during the incubations, PCoA was performed on all samples (Figure 3A). The separation of samples along the first axis, explaining 45% of the variance, indicated a change of the DOM composition over the incubation time. In general, analytical triplicates of each sample were located in close proximity (grey background in Figure 3), indicating good reproducibility of the FT-ICR-MS measurements. The second axis (principal component 2, PC 2) separated the samples of the different sediment depths in two distinct groups, indicating characteristic DOM compositions in both sediment layers. Restricting the PCoA to samples from the individual depths exhibited a more detailed view on changing DOM compositions over the course of the experiment (Figure 3B,C). For the surface incubations, the shift in the DOM composition for the treatment was reflected on the first axis (PC 1 = 55% variance), while changes in the control incubation emerged on the second axis, only. Typical biomolecules (e.g., unsaturated compounds) continuously decreased in relative abundance from 45% to 20% and 29% to 15% in treatment and control, respectively. Accordingly, compounds rather difficult to degrade increased in relative abundance (e.g., highly unsaturated compounds) from 37% to 52% and from 49% to 52% in treatment and control, respectively, indicating an on-going microbial transformation of DOM during the incubation (Supplementary Table S2).

Similar to the DOM samples from the surface, those from 20 cmbsf revealed the strongest separation along the PC 1 axis (52% variance) between day 0 and day 6 (Figure 3C). Accordingly, typical biomolecules decreased (e.g., unsaturated compounds from 34–37% to 19–23%), while more refractory compounds increased in relative abundance (e.g., aromatic compounds from 11–14% to 19–21%). In comparison to the surface incubations, all samples were more similar to each other, indicating a less intense DOM transformation. The most pronounced change in the DOM composition occurred at day 6 of the treatment. At this time point, a variety of typical biomolecules increased, coinciding with the prophage induction event (Figure 1).

### 3.3. Virus-Induction in the 20 cmbsf Slurries Released Transient and Persistent DOM Components

**Figure 4:**
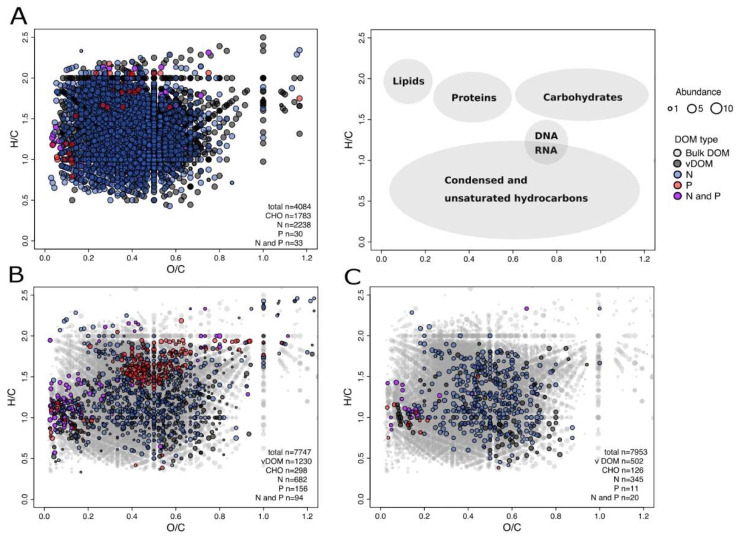
Distribution of the detected DOM molecular formulas over the course of the experiment (day 0 until day 55) of the mitomycin C treated slurry of the 20 cmbsf incubation according to the H/C and O/C ratios (van Krevelen diagram). Each dot represents at least one molecular formula, color-coded according to the N- and P-content and locations of typical molecular compound classes [57] are indicated (see legend). (**A**) Background DOM compounds, identified at day 0, (**B**) freshly released DOM compounds after virus-mediated cell lysis on day 6 (vDOM) and (**C**) remaining vDOM compounds at the end of the experiment are displayed. In B and C, background DOM compounds that were not associated with the virus-induced cell lysis are presented in grey.

The microbial transformation of the freshly produced organic material that was released at day 6 in the treatment of the 20 cmbsf incubation is visualized in van Krevelen diagrams (Figure 4). Symbols representing elemental ratios of O/C and H/C for each molecular formula are color-coded by their respective N and P content. Depending on the position in the van Krevelen diagrams, the molecular formulas can roughly be classified according to major biomolecular components (58). As millions of constitutional isomers can share the same molecular formula, the position in the van Krevelen diagram suggests potential structural features of the DOM composition only and classification is not exclusive (Figure 4 Legend).

At day 0, 4084 individual molecular formulas were detected in the treatment, among others 56% N-, 10% S- and 1.5% P-containing compounds (Figure 4A). At day 6, 1230 new molecular formulas (accounting for 16% of the total) were identified in the treatment that were not present in the corresponding control nor at the beginning of the experiment in both, treatment and control (Figure 4B). Considering that the same abiotic processes (e.g., sorption and desorption on the sediment matrix) took place in both setups, the results suggest that these compounds originated from released cell material following viral lysis (vDOM). It was found that 63% of the compounds contained the heteroatoms N, 28% S and 20% P. These compounds spread out over the whole van Krevelen diagram, indicating the release of a wide array of molecular groups with different biological availabilities.

The inventory of vDOM compounds changed over the course of the experiment. We found that 58% of the identified molecular formulas were no longer detectable after 55 days of incubation (Figure 4C). Preferentially potential biomolecules, e.g., unsaturated compounds, were removed, suggesting that the labile DOM fraction was turned over by bacteria. Particularly, P-containing molecules dropped to 2% of relative abundances of the total molecular formulas. According to the position in the van Krevelen diagrams, the H/C and O/C ratios of consumed compounds fall in the range typical for peptides, amino sugars and carbohydrates (Figure 4 Legend). The remaining material clusters in the area of H/C and O/C ratios matching condensed and unsaturated hydrocarbons, which are less susceptible to microbial degradation and potentially contribute to the pool of refractory DOM.

### 3.4. Prophage Induction Shaped the Bacterial Community Composition

Illumina sequencing was used to determine the development of the microbial community compositions for treatment and control of both investigated depths over the course of the experiment. While sequencing of 16S rRNA genes assessed the present community members, the potentially active community was analyzed by targeting 16S rRNA transcripts. Sequencing resulted in a total of ~790,000 reads with ~17,000 to 30,000 reads for DNA (average: ~24,000 reads) and ~11,000 to 40,000 for RNA samples with an average of ~20,000 reads. The sequences were affiliated to ~39,000 different OTUs on a 97% sequence identity level. On average, the individual DNA samples contained ~2700 and the RNA samples ~1800 OTUs. As an exception, less than 300 OTUs were generated for three RNA samples from ~11,000 to ~16,000 reads, respectively.

**Figure 5:**
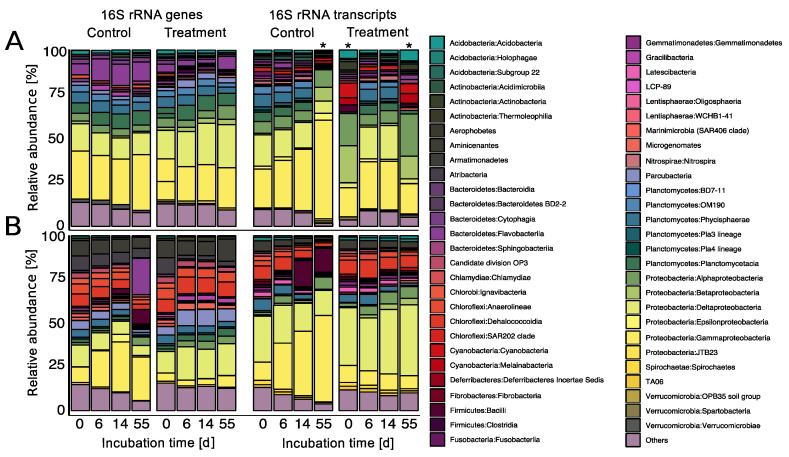
Relative abundances of individual taxa on class level after mitomycin C treatment compared to the non-treated controls (**A**) in the surface slurries and (**B**) 20 cmbsf slurries. DNA-based community structures are displayed on the left, RNA-based community structures on the right, both over the course of the experiment. Taxa with an average abundance <1% throughout the experiment were summed up to “Others”. Samples with identified OTUs <300 are marked with an asterisk (*).

In general, the present (DNA-based) and potentially active (RNA-based) communities show similar patterns in their composition on class level (Figure 5). Proteobacteria was the most predominant phylum in all slurries with abundances ranging between 18 and 59% in the present as well as 39 and 86% in the potentially active communities, with main contributions of Gammaproteobacteria, Deltaproteobacteria and Alphaproteobacteria. However, both depths exhibited characteristic community structures, indicating distinct indigenous communities. While Planctomycetes were the second most abundant (13–23%) and potentially active group (up to 21%) in the surface slurries, Chloroflexi made up a large part of the communities in the 20 cmbsf slurries (3–18%). Relative abundances of Deltaproteobacteria in the 20 cmbsf incubations ranged between 10 and 40%.

In the surface incubations, no major changes in the composition of the present community were observed in both, the control and treatment over the course of the experiment (Figure 5A). For the potentially active community, Gammaproteobacteria slightly increased in the control incubation, most pronounced at day 55. However, this sample contained only 296 OTUs, presumably leading to an overestimation of dominant OTUs. The other two samples with a low number of OTUs also showed a divergent pattern in comparison to all other samples from the surface incubations. Generally, the diversity in terms of OTU numbers of the surface incubations continuously decreased over the course of the experiment as reflected in the declining evenness over time (Table 1).

In the 20 cmbsf incubations, the increase of Gammaproteobacteria within the controls was even more pronounced (Figure 5B). It was not only visible in the potentially active (increase from 10% to 49%), but also in the present community (from 9% to 29%). This shift was accompanied by a strong increase of Firmicutes and Flavobacteria from day 14 on. Associated with the general community shift in the controls, phyla with less than 1% of relative abundance (“Others”) strongly decreased. The increasing predominance of individual groups is mirrored in a drastic decrease of diversity and evenness (Table 1). On DNA level for example, diversity dropped from ~2700 to ~1500 OTUs and evenness from 0.86 to 0.59.

In contrast, the overall community compositions of the treatment from 20 cmbsf only changed marginally over time. The rather stable community composition in the treatment was also reflected in the constant diversity and evenness values on the DNA level with OTU numbers between ~2500 and ~2700 and evenness between 0.84 and 0.89. The OTU numbers of the potentially active community members were halved between day 6 and 14, but the evenness declined from 0.84 to 0.75, only, suggesting that prophage induction resulted in a more diverse and stable community composition compared to the control.

## 4. Discussion

### 4.1. The Productivity of the Bering Sea Supports High Numbers of Benthic Bacteria and Viruses

The high primary production in the surface ocean of the Bering Sea [26] (Supplementary Table S1) fuels the deep-sea ecosystem through particle export from the photic zone, supporting benthic prokaryotes [58]. This is mirrored in the relatively high TCCs and VLP numbers compared to typical deep-sea sediments [4]. While the TCCs are rather similar to those of very productive coastal sites [59], virus numbers were in the same range as previously reported for deep-sea surface sediments with elevated organic carbon concentrations [60]. The drop in prokaryotic and virus abundances from the surface to 20 cmbsf is accompanied by a decrease in the amount and quality of DOM. At this depth, increased ammonium indicates intensively processed organic material (Supplementary Table S1), consistent with higher percentages of more degraded DOM compounds that were already present at the start of the experiment.

### 4.2. Methodological Considerations

#### 4.2.1. Experimental Setup

Due to the described limitations in recovering a sufficient amount of sediments, the incubations could not be set up in replicates. This was mainly due to restrictions in ship time, difficulties in the accessibility of deep-sea sediments and the high demand of sediment volume for the analysis of each parameter. Thus, we decided to use a total of 800 mL of slurry, as for every main sampling day, 165 mL had to be taken. An initially smaller volume would have changed the system’s stability within the incubation bottles over the course of the experiment. Therefore, the results cannot be statistically verified, but as the data set is consistent in itself, we are confident about the interpretation of general trends.

#### 4.2.2. Potential Effects of the Mitomycin C Treatment

In addition to prophage induction, the treatment with mitomycin C can have various potential effects on prokaryotic communities and thus the cycling of DOM, which might partially explain some of the observed phenomena in the experiment. While mitomycin C was used as an effective inducing agent in many studies on lysogeny [61], it only has the potential to induce a limited fraction of prophages. Depending on the study, the percentage of prokaryotes containing mitomycin C inducible prophages was found to range between undetectable to >80%, with several studies reaching an induction rate of ~40% [11]. Therefore, the absolute number of temperate viruses is most likely underestimated when using mitomycin C. Despite this limitation, we assume that the response of prophages to mitomycin C is similar within highly diverse prokaryotic communities. In the current study, the investigated sediment layers contained cell numbers in the range of ~10^8^ and ~10^9^ cells per mL slurry, comprising ~2700 and ~3000 OTUs. Thus, we assume that, a comparable fraction of bacteria was susceptible to mitomycin C induction in both depths.

As mitomycin C inhibits DNA replication and generally can be toxic to microorganisms [62], one might argue that the observed stabilization of the bacterial community composition was due to the treatment with the antibiotic itself. However, as stated above, mitomycin C does not affect all community members of an environmental sample, harboring thousands of different species [61]. Since the TCCs and the number of abundant and potentially active phylotypes in the 20 cmbsf treatment did not drastically collapse compared to the corresponding control, it is unlikely that the antibiotic killed or inhibited a substantial part of the bulk bacterial community. Additionally, we obtained sufficient amounts of intact rRNA for next generation sequencing that were otherwise considerably degraded until day 55 [63], suggesting the presence of metabolically active and diverse bacteria until the end of the experiment.

A potential inhibitory effect of mitomycin C on the prokaryotic metabolism could have influenced the use of labile organic substrate, and thus explain the observed discrepancy between control and treatment (Figure 2). However, an inhibition by mitomycin C would also affect the treated surface community in a similar way, which was not observed in the analyses of amino acid and carbohydrate concentrations. The rapid removal of P-containing compounds in the 20 cmbsf incubations over time (Figure 4C) could have been caused by a specific requirement for the synthesis of nucleic acids. The treatment with mitomycin C might lead to a cellular SOS response, as the antibiotic causes interstrand DNA cross-links [64]. Such a cell response could result in the up-regulation of genes involved in cell repair and maintenance, and increased requirement of phosphorous [8,64]. However, other induction treatments (e.g., UV irradiation and starvation) were not suitable for the described experimental setup (e.g., turbidity of the slurries and deep-sea communities adapted to low substrate availability). Further, the removal of mitomycin C by washing steps would have dramatically disturbed the DOM and bacterial community compositions.

### 4.3. The Accumulation of Labile Organic Compounds Infers a Reduced Metabolic Potential of the Communities from 20 cmbsf

In the surface slurries, the constantly low carbohydrate and amino acid concentrations throughout the incubation indicate a metabolically versatile bacterial community, which efficiently consumes the available substrates. The increasing DOC, DCAA and DCCHO concentrations in the 20 cmbsf incubations (Figure 2) might be explained by the generally lower cell numbers (Figure 1A) and the drop in diversity (Table 1), associated with a diminished metabolic activity, resulting in less efficient utilization of available substrates [65]. A rather inactive community would also explain the initially higher concentrations of labile organic carbon compounds and comparably smaller changes in the DOM composition in the 20 cmbsf incubations over time (Figure 3C). The constantly rising DOC concentrations (Figure 2A) reflect an accumulation of refractory compounds during the incubation [66]. The increase of labile organic components (Figure 2B–E) might point towards a release of organic material from the sediment matrix due to the slurry process [67], as observed before [68]. Amino acid and carbohydrates contributed only marginally to the DOC pool, indicating that the bulk of the released organic carbon compounds remained uncharacterized (Supplementary Table S3).

The observed discrepancy in organic carbon compound concentrations between the control and treatment of the 20 cmbsf incubations could be a result of different community compositions in the treatment and control. The control showed a community shift to more copiotrophic Gammaproteobacteria and Flavobacteria, which are known to be better adapted to high substrate concentrations [69]. This might partially explain the better exploitation of organic compounds in the controls compared to the treatment.

### 4.4. Viral Lysis and Subsequent Microbial Processes Were Imprinted in the DOM Composition

The main changes of the DOM molecular composition were observed along the time axis of the experiments (Figure 3). The decomposition of bulk organic compounds and molecules that were probably liberated by slurrying led to a gradual removal of bio-available components, accompanied by a relative accumulation of more refractory compounds [66,70]. Despite the clear alteration of the DOM composition by the slurry process, DOM analysis revealed that prophage induction had modified the chemical composition and character of the benthic DOM pool, which is in line with previous studies [12,20,71]. During virus-mediated cell lysis, the chemical complexity of DOM increased and resulted in the generation of compounds that were compositionally distinct from the bulk DOM. However, our definition of freshly produced material is rather conservative, most likely underestimating the overall amount of vDOM compounds. On the one hand, virus-induced cell lysis may also have occurred naturally in the controls or in the beginning of the experiment, e.g., during core recovery or setting up the slurries [10], leading to the exclusion of overlapping formulas from the vDOM. On the other hand, only a certain fraction of these lysis products can be observed in the analytical window of the applied methods [72,73,74].

In general, the virus lysate was characterized by an increased fraction of heteroatom-containing formulas and unsaturated compounds compared to the initial and bulk DOM composition of the treatment and the corresponding control (Supplementary Table S2) [57]. Increased average molecular masses and numbers of carbon atoms [75] as well as the oxidation and saturation state [76] indicated diagenetically younger, freshly produced material. The chemical characteristics of the vDOM identified in our study are comparable to viral lysis products of a previous analyzed *Synechococcus* strain, as identified by similar positioning of compounds in the van Krevelen plots [71]. While every vDOM signature is most likely defined by the specific cellular composition of the analyzed microorganisms (e.g., phycobilisome components of the *Synechococcus* lysate [71]), our results point towards a rather uniform molecular nature of bulk DOM signatures of released cell material [71] and virus particles [77].

Roughly 60% of the released compounds were selectively removed until the end of the experiment, most of which can be assigned to biomolecules (Figure 4C). This is in accordance with other studies, showing that labile lysis products and virus particles were quickly metabolized, providing organic compounds and key elements to heterotrophic microorganisms [5,19,78]. In contrast to previous observations, N-containing compounds rather increased during the experiment [12]. The degradation of these molecules might have been masked by a constant input of amino acids and peptides due to leaching from the slurries.

The strong enrichment of phosphorous-containing compounds following prophage induction indicated the release of cell components, e.g., nucleotides or phosphorylated intermediates [79], and virus particles, which are enriched in P compared to prokaryotic cell debris [77]. The rapid removal of P-containing molecules was also observed in previous studies [5,77,78]. Furthermore, the assembly of new virus particles implies a high P-demand, usually derived from the host’s nucleotides [80] or from extracellular resources [81], which is particularly necessary in P-limited ecosystems. There is evidence for high turnover rates of P-species with increasing water depths close to our sampling station [82].

At the end of the experiment, the treatment was still enriched in potential biomolecules, probably of a more persistent nature that could eventually contribute to the global inventory of refractory DOM (Figure 4C). The relative intensities of these remnant molecular formulas increased over time, suggesting preferential accumulation or microbial production of more resistant molecules [66]. Considering the slow metabolism of deep-sea microorganisms [65], the incubation period of 55 days was probably too short for the complete degradation of more complex compounds. Overall, our findings highlight the importance of viral lysis for the rapid regeneration of nutrients and the provision of bioavailable DOM to microorganisms inhabiting an environment where most DOM is refractory and bio-available compounds are scarce.

### 4.5. Prophage Induction Indicates a Predominance of Temperate Viruses in 20 cmbsf

Initially, we expected prophage induction in the treatments of both investigated depths. Prophage induction was defined by a considerable increase of the VCR in the treatment compared to the control to exclude non-related effects, e.g., the death of prokaryotic cells due to the antibiotic. Surprisingly, the treatment of 0 cmbsf showed no increase in VCRs, while increasing VCRs in the 20 cmbsf treatment are a result of a weak decline of TCCs combined with a simultaneous rise of VLP numbers. The discrepancy of the VCRs at the surface and the 20 cmbsf samples can be interpreted as a prevalence of the lytic lifestyle at the surface of deep-sea sediments, supporting the findings by Danovaro et al. [4]. The comparably late induction in our study can be related to the overall low turnover rate of the deep-sea microbial community [65] and the low in-situ incubation temperature. While in laboratory cultures, prophage induction usually occurs within a couple of hours after mitomycin C treatment, Zhao et al. [12] also reported that an isolated cyanophage took five days to completely lyse a *Synecococcus* culture. In our experiment, the observed drop of cell numbers from the surface to the 20 cmbsf incubations was probably caused by a decreasing availability of suitable substrates [1]. This is reflected in the initially high concentration and further accumulation of organic material within the 20 cmbsf slurries (Figure 2). The change in activity of host cells and quality of DOM below the seafloor could have led to a shift in the virus lifestyle towards lysogeny. This finding aligns with studies linking the lytic cycle to high host’s densities and metabolic activities, while lysogeny is assumed to be favored under harsher environmental conditions [11]. The prevalence of the lysogenic lifestyle further extends in much deeper sediment layers [83,84], where increasing intercell distances decrease the probability of a virus particle to encounter its next host. Thus, the shallow subsurface of Bering Sea sediments at 20 cmbsf might represent an intermediate state for the switch from lytic to lysogenic dominated communities.

### 4.6. Prophage Induction Stabilized Bacterial Community Composition According to the “Killing the Winner” Model

The initial bacterial community structures of both depths were similar to those found in other studies on deep-sea sediments [85]. In our incubations, the experimental manipulation had a strong influence on the community composition of the controls (Figure 5). The so-called “bottle effect” [86] favored the growth of copiotrophic Gammaproteobacteria and Flavobacteria, while less abundant phyla (<1%) decreased in relative abundances. In marine environments, both bacterial groups have for example been found to rapidly respond to diatom blooms [87]. Considering the annual recurring algal blooms in the Bering Sea and the olive-grey diatomaceous silt of the slurries [88], these groups could have grown in the incubations, degrading the buried algal biomass within the slurries. Consistently, the observed bottle effect was more pronounced in the 20 cmbsf control, where the amount of labile organic matter was higher than in the surface incubations (Figure 2A). The copiotrophic groups were likely better adapted to the substrate-rich conditions in the experimental setup and thus were able to outcompete less specialized, slower growing microorganisms [87]. To a lesser extent, this can also be seen in the control of the surface incubation. Unfortunately, only ~11,000 reads and ~300 OTUs were detected in the RNA sample from day 55. Thus, rare taxa might have been discriminated and are therefore underrepresented in the data set.

In contrast to our previous expectations, the treatment of 20 cmbsf showed a remarkably stable community composition over the course of the experiment (Figure 5B). We relate this stabilization to the prophage induction event; a scenario consistent with the “killing the winner” model [21,89]. This effect on the community structure is reflected in higher diversity, richness and evenness within the treatment from 20 cmbsf (Table 1). For the treatment of the surface incubation, RNA samples from day 0 and day 55 contained ~16,000 and ~12,000 reads, both resulting in OTU numbers below 300, only. The lower quality of the sequencing data again interferes with a sound interpretation of the community dynamics. The observed community stabilization of the treatment of 20 cmbsf somewhat contradicts the outcome of an experiment in which a pelagic microbial community was amendment with vDOM from a cyanobacterial culture [12]. There, the community composition changed, and diversity increased in response to the addition of viral lysis products. This study is only in parts comparable to our investigation as the lysis of selected community members is not taken into account in the pelagic study. A similar stabilization in diversity, richness, and evenness as in this experiment was observed in a study using a virus reduction approach [90]. There, abundances of copiotrophic groups were controlled by viruses at elevated DOM concentrations. The authors concluded that the degree of viral regulation on population dynamics depends, among other factors, on the concentration of DOM. To draw more general conclusions on the effect of viruses on community compositions, further experiments are needed with deep-sea sediments of different trophic backgrounds.

## 5. Conclusions

The indigenous bacterial populations and the DOM pool were influenced by viral induction, depending on the depth of the sediment. While no prophage induction was observed in samples from the seafloor, a higher proportion of viruses in 20 cmbsf was lysogenic compared to the sediment surface. This shift to the lysogenic cycle at greater sediment depth is probably linked to decreasing abundances of suitable hosts. The induction event resulted in a stabilization of bacterial diversity, equalizing the typical bottle effect observed in the control. Thus, in deeper sediment layers, temperate viruses could play a crucial role in regulating the system’s stability. Additionally, virus-mediated cell lysis provided labile organic compounds that were effectively recycled by the resident bacterial community, leaving behind more refractory DOM components that potentially accumulate in the sediments.

## Figures and Tables

**Table 1:** Changes in the diversity indices for the bacterial communities of the 0 and 20 cmbsf slurry incubations, calculated from the libraries of 16S rRNA genes (DNA) and transcripts (RNA) over the experimental period.

Incubation			Day	No. of OTUs	Shannon Diversity Index	Pielou’s Evenness
**0 cmbsf**	DNA	Control	0	3019 ± 27	6.9 ± 2.6	0.93 ± 0.13
			6	2956 ± 29	6.8 ± 2.6	0.91 ± 0.12
			14	2817 ± 24	6.7 ± 2.4	0.90 ± 0.12
			55	2691 ± 28	6.8 ± 2.6	0.89 ± 0.12
		Treatment	0	2962 ± 25	7.0 ± 2.6	0.93 ± 0.12
			6	3153 ± 22	7.0 ± 2.4	0.94 ± 0.12
			14	2928 ± 23	6.8 ± 2.5	0.92 ± 0.13
			55	2642 ± 29	6.3 ± 2.2	0.86 ± 0.12
	RNA	Control	0	3503 ± 19	7.1 ± 2.1	0.95 ± 0.13
			6	3426 ± 19	7.0 ± 2.3	0.94 ± 0.13
			14	2947 ± 21	6.4 ± 2.1	0.86 ± 0.11
			55	296 ± 0 *	4.4 ± 0	0.59 ± 0.08
		Treatment	0	123 ± 2 *	3.9 ± 0	0.54 ± 0.09
			6	3615 ± 17	7.0 ± 2.2	0.94 ± 0.13
			14	3020 ± 22	6.7 ± 2.2	0.91 ± 0.13
			55	247 ± 1 *	4.7 ± 0	0.62 ± 0.08
20 cmbsf	DNA	Control	0	2702 ± 25	6.4 ± 1.9	0.86 ± 0.12
			6	2455 ± 25	6.0 ± 1.8	0.81 ± 0.12
			14	2131 ± 26	5.5 ± 1.3	0.73 ± 0.10
			55	1500 ± 20	4.4 ± 0.8	0.59 ± 0.08
		Treatment	0	2747 ± 22	6.4 ± 2.3	0.87 ± 0.12
			6	2605 ± 22	6.6 ± 2.2	0.89 ± 0.11
			14	2629 ± 25	6.7 ± 2.4	0.89 ± 0.12
			55	2465 ± 18	6.3 ± 2.0	0.84 ± 0.11
	RNA	Control	0	2261 ± 22	6.4 ± 2.2	0.84 ± 0.09
			6	1986 ± 19	5.7 ± 1.5	0.76 ± 0.1
			14	1490 ± 18	4.7 ± 0.8	0.63 ± 0.09
			55	792 ± 12	3.7 ± 0	0.50 ± 0.07
		Treatment	0	1943 ± 17	6.2 ± 2	0.83 ± 0.11
			6	1940 ± 22	6.2 ± 2.1	0.84 ± 0.12
			14	973 ± 9	5.6 ± 1	0.75 ± 0.09
			55	833 ± 5	5.4 ± 0.7	0.75 ± 0.11

No. = number. Samples with identified OTUs < 300 are marked with an asterisk (*).

## Supplementary Materials

The following are available online at https://www.mdpi.com/1999-4915/12/9/922/s1, Table S1: Geochemical composition of bulk sediment and pore water from 0 and 20 cmbsf used for the slurry incubations, Table S2: Comparison of all FT-ICR-MS spectra, including the virus-mediated cell material (vDOM) and analytical standards (NEqPiW), Table S3: Contribution of total hydrolysable amino acids (THDAA) and total hydrolysable dissolved monosaccharides (THCHO) to bulk dissolved organic carbon concentrations.
