# Supplementary material for: Impact of Viral Lysis on the Composition of Bacterial Communities and Dissolved Organic Matter in Deep-Sea Sediments
Source: Viruses. 2020 Aug 22;12(9):922. doi: 10.3390/v12090922 (PMC7552059; doi:10.3390/v12090922)
Supplement: Supplementary file 1 [file viruses-12-00922-s001.pdf]

Supplementary material

Impact of viral lysis on the composition of bacterial communities and dissolved organic matter in deep-sea sediments

Mara E. Heinrichs[1†], Dennis A. Tebbe[1†], Bernd Wemheuer[2], Jutta Niggemann[1], Bert Engelen[1*]

[1] Institute for Chemistry and Biology of the Marine Environment, Carl von Ossietzky University of Oldenburg, 26129 Oldenburg, Lower Saxony, Germany

[2] Genomic and Applied Microbiology and Göttingen Genomics Laboratory, Institute of Microbiology and Genetics, University of Göttingen, 37073 Göttingen, Lower Saxony, Germany

[†] These authors contributed equally to this work

* Correspondence: engelen@icbm.de Tel.: (+ 49 441 798 5376)

This file includes:

Tables S1 to S3

Table S1. Geochemical composition of bulk sediment and pore water from 0 and 20 cmbsf used for the slurry incubations: pore water nutrient concentrations of ammonium (NH_4), nitrite (NO_2), nitrate (NO_3), phosphate (PO_4) and silicic acid (Si); sediment total organic carbon (TOC), carbon (C) and sulfur (S) contents. Sea surface Chlorophyll a concentration 119.28 mg / cm^3

Parameter	Sediment depth	
	0 cmbsf	20 cmbsf
NH_4 [μM]	13.5	109.9
NO_2 [μM]	- *)	-*)
NO_3 [μM]	30.5	-*)
PO_4 [μM]	3.0	12.5
Si [μM]	338	367
TOC [%]	1.25	1.19
Total C [%]	1.3	1.25
Total S [%]	0.28	0.17

*) Below quantification limit of the method.

Table S2. Comparison of all FT-ICR-MS spectra, including the virus-mediated cell material (vDOM) and analytical standards (NEqPiW). Considered are only peaks with assigned molecular formulae, identified in all replicate analyzes of the samples (n=2-3) and analytical standards (NEqPiW, n=36) (weighted by FT-ICR-MS peak intensity).

	**0 cmbsf**								**20 cmbsf**									
	**Treatment**				**Control**				**Treatment**				**Control**				**NEqPiW**	**vDOM**
	**D0**	**D6**	**D14**	**D55**	**D0**	**D6**	**D14**	**D55**	**D0**	**D6**	**D14**	**D55**	**D0**	**D6**	**D14**	**D55**		
No. of peaks	3351	7018	6577	5274	6013	3604	6059	6285	4084	7747	6323	7953	5905	7251	7280	7056	3398	1230
Av. m/z of peaks	335	371	367	349	361	322	361	351	344	379	359	370	362	370	369	368	433	434
Av. C	16.82	17.29	16.96	16.56	16.98	15.62	16.67	16.80	16.72	17.76	16.87	17.35	17.55	17.40	17.28	17.21	20.77	19.97
Av. H	24.85	21.60	20.04	20.09	22.34	20.04	19.15	19.50	23.53	22.53	21.66	21.44	23.92	21.64	21.54	20.81	26.28	25.38
Av. O	5.82	7.54	7.62	6.94	7.24	6.22	7.53	6.80	6.48	7.56	7.11	7.38	6.81	7.39	7.46	7.51	8.71	8.40
Av. N	0.95	1.11	1.14	1.08	0.99	0.94	1.06	1.10	0.97	1.17	1.16	1.20	1.04	1.14	1.11	1.07	0.80	1.35
Av. S	0.07	0.16	0.17	0.14	0.16	0.08	0.18	0.16	0.10	0.17	0.15	0.19	0.13	0.16	0.18	0.19	0.16	0.32
Av. P	0.02	0.03	0.04	0.03	0.02	0.01	0.04	0.05	0.02	0.05	0.02	0.02	0.02	0.02	0.02	0.02	0.08	0.20
Av. H/C	1.43	1.24	1.18	1.20	1.30	1.27	1.14	1.6	1.40	1.28	1.29	1.24	1.36	1.25	1.25	1.21	1.26	1.31
Av. O/C	0.37	0.46	0.47	0.43	0.44	0.41	0.47	0.43	0.40	0.44	0.44	0.44	0.40	0.44	0.45	0.45	0.43	0.47
Molecular indices																		
Av. AI_{mod}	0.21	0.27	0.31	0.31	0.25	0.29	0.33	0.35	0.22	0.26	0.26	0.28	0.24	0.28	0.28	0.30	0.26	0.23
Av. DBE	5.88	8.06	8.53	8.07	7.32	7.07	8.65	8.63	6.44	8.11	7.63	8.24	7.11	8.16	8.07	8.34	9.07	9.06
% Aromatics	12.7	18.3	22.1	22.9	16.4	20.3	24.8	27.4	11.3	16.3	16.5	19.3	14.1	19.0	19.0	20.8	81.6	14.8
% Highly unsat.	37.0	53.8	56.4	52.0	49.3	49.2	56.7	51.9	45.4	53.1	51.0	52.5	46.5	52.5	52.0	54.8	7.2	50.2
% Unsaturated	45.3	23.1	16.5	19.9	28.9	24.7	13.2	15.4	37.6	25.4	26.9	23.0	33.9	23.3	23.9	19.4	0.5	29.9
% Saturated	0.5	0.3	0.3	0.1	1.0	0.4	0.2	0.5	1.0	0.6	0.5	0.4	1.0	0.4	0.3	0.1	0.5	1.5
% Saturated N	0.2	0.1	0.2	0	0.2	0.1	0.1	0.1	0.2	0.3	1.0	0.1	0.2	0	0	0	55.4	1.4

Av. = average, No. = number, AI_{mod} = modified aromaticity index, DBE = double bond equival

Table S3. Contribution of total hydrolysable amino acids (THDAA) and total hydrolysable dissolved monosaccharides (THCHO) to bulk dissolved organic carbon concentrations in the 0 and 20 cmbsf slurries during incubation time (day 0-55). Percentages are calculated on carbon basis.

	**Sample**	**% THDAA**				**% THCHO**			
		**D0**	**D6**	**D14**	**D55**	**D0**	**D6**	**D14**	**D55**
0	Control	4.5	1.6	1.7	1.8	3.3	2.2	3.5	2.9
cmbsf	Treatment	3.5	2.0	2.3	2.1	3.1	2.4	2.5	3.6
20	Control	6.5	5.0	4.9	5.0	10.2	7.8	8.7	9.2
cmbsf	Treatment	6.1	5.8	6.5	7.5	8.6	7.0	7.4	7.8

Day 0 – 55 (D0 – D55)
